# Sleep Apnea and Atrial Fibrillation; 2012 Update

**DOI:** 10.2174/157340312803760811

**Published:** 2012-11

**Authors:** Genevieve C Digby, Adrian Baranchuk

**Affiliations:** Department of Cardiology, Kingston General Hospital, Queen's University, Kingston, Ontario, Canada

**Keywords:** Atrial fibrillation, sleep apnea, obstructive sleep apnea.

## Abstract

Atrial fibrillation (AF) and obstructive sleep apnea (OSA) are very prevalent diseases in modern society. Recent years have seen the emergence of a wide body of literature suggesting an important association between these two diseases. This review will provide a summary of this evidence as it currently exists. First, it will review the literature suggesting an association between AF and OSA by highlighting the prevalence of AF in OSA, the correlation of AF prevalence with OSA severity and the trend towards increased AF recurrence in patients with OSA after treatment for AF. Second, it will identify the possible pathophysiologic mechanisms for this association. In doing so, it will discuss the investigated effects of intrathoracic pressure changes, autonomic instability and atrial remodeling. Finally, it will review the evidence of the effect of treatment of OSA on AF, highlighting the role of continuous positive airway pressure (CPAP) in the treatment of OSA and its impact on AF prevalence and recurrence.

## INTRODUCTION

Atrial fibrillation (AF) is the most common sustained arrhythmia in the US, affecting approximately 2.3 million people and accounting for one third of all cardiac rhythm hospitalizations [1]. It is estimated that individuals 40 years of age and older have a lifetime risk of one in four of developing AF [2] and that 5.6 million people in the US will be affected by 2050 [3]. The clinical impact of AF results primarily from its association with stroke, heart failure, and increased overall mortality [4,5]. Many conditions may also predispose an individual to the development of AF, including hypertension, coronary artery disease, valvular heart disease, congestive heart failure (CHF), and obesity [1]. There is also increasing evidence of an important association between sleep apnea and AF [6,7].

Obstructive sleep apnea (OSA) is a common sleep-related breathing disorder (SBD), affecting an estimated 5% of the North American adult population [8] and associated with an increased risk of cardiovascular disease [9]. OSA is characterized by repetitive occlusions of the upper airway during sleep, the physiological consequences of which include arterial hypoxemia and hypercapnia, endothelial dysfunction, and sympathetic activation, which in turn lead to an increased risk of cardiovascular morbidity and mortality [10]. It is associated with systemic and pulmonary hypertension, coronary artery disease, CHF, and arrhythmias, as well as daytime hypersomnolence, and neurocognitive dysfunction [11-16]. Additionally, OSA is an independent risk factor for stroke, and it may increase stroke risk in patients with AF [1,17]. The gold standard for diagnosis of OSA is over-night polysomnography, during which severity can be characterized by the number of apneas and hypopneas per hour of sleep, the apnea-hypopnea index (AHI). Accepted AHI cut-points of 5, 15, and 30 are used to indicate mild, moderate, and severe OSA, respectively [18,19].

In the last few years, there has been mounting evidence for an important association between AF and OSA. In fact, the prevalence of OSA among patients with AF is estimated to be about 32%–49% [20]. Concurrently, there is a growing body of literature that attempts to identify the mechanisms of the interactions between these conditions. 

This review will attempt to summarize this evidence. First, we will review the literature suggesting an association between AF and OSA. Second, we will identify the possible pathophysiologic mechanisms for this association. Finally, we will review the evidence of the effect of treatment of OSA on AF. 

## ASSOCIATION OF AF AND OSA

The evidence reporting an association between AF and OSA is expanding exponentially. The earliest description of an association between AF and OSA emerged from epidemiologic studies. Subsequently, evidence suggested that severity of OSA correlated with prevalence of AF. Recently, this association has been shown to apply to a vast number of subgroups of patients with OSA. The evidence is reviewed below.

### Prevalence of AF in OSA

Several observational studies reported an association between cardiac arrhythmias and OSA [21-23]. One of the first studies to clearly delineate the prevalence of AF in OSA emerged from patients studied in the 1970’s. In this investigation, Guilleminault *et al* [21] performed 24-hour Holter monitoring on 400 patients with moderate-to-severe OSA (AHI ≥ 25) and found a 3% prevalence of AF. This reported prevalence of AF is more than three times the prevalence within the general population which is estimated at 0.4-1.0% (24). More recently, the Sleep Heart Health Study [25], a large cross-sectional study assessing the prevalence of cardiac arrhythmias in 228 individuals with SBD (respiratory disturbance index (RDI) ≥ 30/h) compared to 338 individuals without SBD (RDI ≤ 5/h), demonstrated a significantly higher prevalence of AF in individuals with SBD (4.8% versus 0.9%, p = 0.003). It is important to note, however, that this study did not differentiate central sleep apnea from OSA. Another study by Gami *et al* [26] retrospectively studied a cohort of 3,542 adults without prior history of AF who underwent polysomnography. They demonstrated that for individuals less than 65 years old, both OSA (AHI ≥ 5) and a decrease in nocturnal oxygen saturation (per 0.5 U log change) were independent predictors of incident AF. This was also the first study to show that OSA and obesity were independent risk factors for incident AF in individuals less than 65 years of age.

Despite the growing evidence of an association between AF and OSA, not all studies have been able to replicate these findings. One study by Flemons *et al* failed to find a relationship between cardiac arrhythmias of any kind and sleep apnea (AHI > 10) [27]. Additionally, one case-control study by Porthan *et al* [28] assessed the prevalence of OSA in 59 patients with lone AF and 56 matched controls and found no difference. Though they found that there was a high prevalence of OSA (AHI ≥ 15) in the lone AF group at 32%, this was not statistically different from the control group where the prevalence of OSA was 29% (p = 0.67). However, given the relatively high prevalence of OSA noted in the control group, some have suggested these discordant results may have been influenced by selection bias [24].

Though not all studies have been able to identify an association between cardiac arrhythmias and OSA, there is undeniably an emerging body of evidence of an association between AF and OSA, and from this has stemmed further evidence of a correlation between severity of OSA and prevalence of AF.

### Correlation Between Severity of OSA and Prevalence of AF

An early study by Hoffstein *et al* [22] prospectively followed 458 subjects undergoing polysomnography for evidence of cardiac arrhythmias. They reported a 58% prevalence of arrhythmia in patients with OSA (AHI ≥ 10) compared to 42% in patients without OSA (AHI ≤ 10) (p <0.0001). They also demonstrated that increased AHI was associated with increased frequency of cardiac arrhythmias (70% of patients with an AHI ≥ 40 versus 42% of patients with an AHI ≥ 10, p = 0.002). However, this study did not report the prevalence of AF separately from other forms of supraventricular arrhythmias, preventing clear associations with OSA from being derived.

Since then, several studies have assessed the prevalence of AF independent of other arrhythmias in SBD, though some variation exists in the definitions of OSA and SBD between studies. For example, a population based study by Tanigawa *et al* [29] examined the association between the frequency of nocturnal oxygen desaturation and prevalence of AF, whereby SBD was defined as a 3% oxygen desaturation index (ODI). In this study, a significant association between the severity of SBD and the prevalence of AF was found, such that the odds ratios for AF were 2.47 (95% CI = 0.91–6.69) for individuals with 5-15 events/hour of 3% ODI level and 5.66 (95% CI = 1.75–18.34) for individuals with ≥ 15 events/hour of 3% ODI level (p for trend = 0.02). This trend suggests that the association between SBD and AF prevalence is related to the extent of hypoxemia and therefore the severity of sleep apnea. 

### OSA as a Predictor of AF in Subgroups

Several studies have assessed the presence of OSA as a predictor of AF in specific subgroups including post-cardiac surgery, post-electrical cardioversion, post-ablation, or in association with underlying CHF [30,34].

Mooe *et al* (30) studied the relationship between OSA and the frequency of post-operative AF requiring pharmacologic or electrical cardioversion. They performed polysomnography in 121 consecutive patients prior to coronary artery bypass grafting surgery and monitored the patients prospectively until discharge from hospital after surgery. They showed that patients with an ODI ≥ 5 had a significantly higher incidence of AF than those with an ODI < 5 (39% vs. 18, p = 0.02). This resulted in a relative risk of AF of 2.8 (95% CI = 1.2–6.8) for those with an ODI ≥ 5 and this was independent of sex, hypertension, and ventricular function. They concluded that pre-operatively diagnosed SBD with nocturnal hypoxemia is an independent predictor of AF after coronary bypass surgery.

In an analysis of the recurrence of AF post-electrical cardioversion, Kanagala *et al* [31] found that the rate of AF recurrence was significantly higher in 27 patients with untreated OSA (AHI ≥ 5) compared to a control group of 79 patients with unknown OSA status (82% versus 53%, p = 0.009). AF recurrence was independent of BMI, age, and hypertension, but correlated with time spent at an oxygen saturation <90%. The results of the study of Kanagala *et al* are consistent with the fact that a significantly higher proportion of patients presenting for AF cardioversion have OSA compared to the general cardiology clinic population (49% versus 32%, p = 0.0004) and this once again highlights a potential association between AF and OSA [32]. 

Meanwhile, a meta-analysis of AF recurrence after catheter ablation by pulmonary vein isolation identified that patients with OSA have a 25% greater risk of AF recurrence after catheter ablation than those without OSA (risk ratio 1.25, p = 0.003) [33].

Finally, in the setting of patients undergoing ablation treatment for AF, several studies have highlighted an increase in recurrence of AF when OSA is comorbid with the arrhythmia. In fact, Bitter *et al* prospectively studied 82 patients with AF undergoing cryoballoon ablation as treatment for AF and found that moderate to severe SBD (AHI ≥ 15) was an independent predictor for AF recurrence post-ablation (hazard ratio = 2.95, p = 0.04) [34].

Interestingly, though OSA has been found to be a predictor of recurrence of AF after successful pulmonary vein isolation, the same many not apply to atrial flutter (AFL). A recent study from our group retrospectively analyzed 122 consecutive patients with right-sided isthmus-dependent AFL referred for ablation and observed that sleep apnea was not a predictor of AFL recurrence (6.1% in patients with SA versus 11.2% in patients without SA, p = 0.39) [35]. It has been speculated that this difference in post-ablation recurrence between in AF and AFL may be that different anatomical targets are involved or that persistence of sleep apnea after AF ablation may predispose new triggers to regenerate AF, while in AFL this may be prevented. Another explanation for this may be that in right-sided AFL, the cavotricuspid isthmus is a crucial part of the circuit and ablation of this structure results in definitive cure of this condition, while in AF, in addition to the triggers within the pulmonary veins, other pathophysiologic mechanisms may also play a role. In fact, interatrial block (IAB) is highly prevalent in patients with both AF and OSA [36-38]. We like to speculate that the high recurrence of AF after pulmonary vein isolation in patients with OSA is not related to reconnections at the pulmonary veins, but may be due to an increased prevalence of IAB in this population. The complete block at the level of the Bachmann bundle may induce interatrial dyssynchrony that could facilitate AF by a different mechanism than the pulmonary veins [36, 38].

One group of patients in which the burden of AF is particularly increased is that of congestive heart failure patients (CHF). Unsurprisingly, a growing body of literature has attempted to address this association in the context of OSA. Javaheri *et al* [39] studied 81 subjects with stable CHF and an ejection fraction less than 45% who underwent polysomnography. They reported that 51% of subjects had some form of SBD, with 40% diagnosed with central sleep apnea (CSA) and 11% diagnosed with OSA. They also noted that subjects with SBD (AHI ≥ 15) had a significantly higher prevalence of AF than those without, 22% versus 5% respectively (p = 0.026), though they did not report the distribution of OSA and CSA within the AF population. A similar study by Sin *et al* [40] retrospectively studied 450 consecutive patients with CHF who were referred for polysomnography and found that patients with CSA had a significantly higher prevalence of AF than those without, 23% versus 11.9% respectively, p <0.05. However, they found no significant difference in the prevalence of AF in OSA patients compared to those without SBD (11.9% and 7.5%). However, these results must be interpreted in caution as they are likely influenced by other etiologies of AF.

There is mounting evidence of an association between AF and OSA and it also appears as though the severity of OSA correlates to a certain extent with the prevalence of AF. This has been shown across various subgroups of patients. It has been hypothesized that SBD, particularly OSA, may lead to AF, though this has not yet been proven. In order to draw such a conclusion, the pathophysiology of AF in patients with OSA needs to be further understood.

## PATHOPHYSIOLOGY OF AF AND OSA

**Fig. (1):**
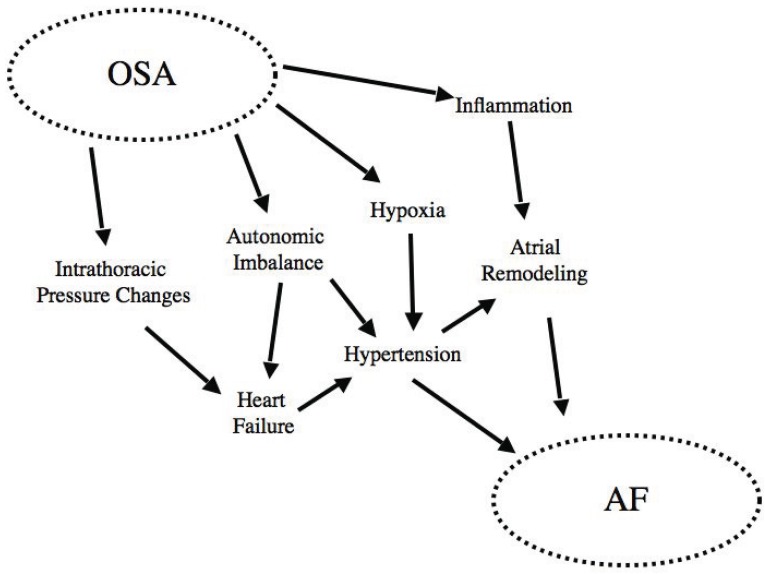
“Interactions of the pathophysiological mechanisms implicated in linking OSA and AF. Reproduced with permission from Todd *et al* [7].

There is undoubtedly increasing evidence of an association between AF and OSA. Though the mechanisms linking OSA with cardiac arrhythmias remain somewhat speculative, there is also mounting evidence of a plausible pathophysiologic link between AF and OSA. Some of these possible links include effects on intrathoracic pressure, impaired autonomic nervous control, and the role of inflammation (Fig. **1**).

### Intrathoracic Pressures Changes

OSA is characterized by repetitive occlusions of the upper airway during sleep that generate substantial shifts in intrathoracic pressure, producing gradients of up to -65 mmHg [41]. These gradients are then transmitted from the thorax to the thin-walled atria and the transmural forces are thought to contribute to atrial chamber enlargement and fibrosis, both known risk factors for AF [24,42]. In addition, it has been suggested that these transmural forces may be important in tissue stretch and remodeling at the pulmonary vein (PV) ostia [24], a known focal source of AF [42]. It has also been suggested that negative tracheal pressure during obstructive respiratory events may be a strong trigger for AF, causing right atrial refractory period shortening and increased susceptibility to AF mainly by enhanced vagal activation [43].

### Instability of Autonomic Tone/Sympathetic Activation

Recurrent nocturnal apneas during sleep in OSA are accompanied by chemoreceptor-induced sympathetic activation and/or decreased parasympathetic tone manifested as impaired vagal input, diminished baroreflex sensitivity, and impairment of the parasympathetic components of heart rate variability [6]. Decreased heart rate variability and increased blood pressure variability have been shown to elevate cardiovascular risk, particularly for heart failure, myocardial infarction, arrhythmic complications, and death, and have even been associated with OSA [44,45]. This heightened sympathetic activity has also been demonstrated to cause peripheral vasoconstriction resulting in elevations in systemic blood pressure [46,47].

Though decreased parasympathetic activation appears to predominate in severe OSA patients, there is evidence of rare increased parasympathetic activation toward the end of apneas in some. This is thought to occur as an oxygen conservation reflex in response to the apnea induced hypoxemia and is mediated through increased vagal tone [25]. This reflex may produce significant bradycardia with a resultant reduction in the refractoriness of the atria, promoting an enhanced susceptibility to discharges from the PV ostia and leading to AF [24].

This difference in autonomic profile has not been consistently reported. A study from our group recently compared levels of autonomic dysfunction between 20 patients with severe OSA (AHI ≥ 30) and 10 patients without OSA (AHI ≤ 5) by measuring heart rate variability (HRV), a non-invasive quantitative technique for assessment of autonomic activity [48]. We found no significant difference in any HRV parameters between patients with severe OSA and controls, and no significant difference in HRV between OSA patients before and after treatment with continuous positive airway pressure. It was speculated that the difference in findings may be related to the studied population and/or difference HRV recording durations. A subsequent study evaluated the relationship between the severity of OSA and heart rate asymmetry (HRA) during sleep [49]. It was found that patients with severe OSA (n = 41; AHI ≥ 30) had significantly reduced deceleration and acceleration runs compared with patients with moderate OSA (n = 18; AHI 5-30) or no or mild OSA (n = 19; AHI ≤ 5), and that this trend correlated with severity of OSA (p < 0.05 for all comparisons). The changes in autonomic profile associated with sleep apnea still need further characterization. 

### OSA and Atrial Remodeling

There is increasing evidence that OSA affects the atrial myocardium, though the mechanisms by which this occurs are still not fully understood. To begin with, an association between OSA and hypertension has been extensively reported [14,50-52]. It has been speculated that a possible link between OSA and AF could be left atrial enlargement that occurs as a result of elevated left ventricular pressures from hypertension induced diastolic dysfunction [6]. Diastolic dysfunction has been shown to be a powerful predictor of left atrium (LA) size [53] and AF [54], and several studies have demonstrated the independent association of diastolic dysfunction and OSA [55-57]. 

Furthermore, IAB (defined as *P*-wave duration ≥120ms) is recognized in the pathogenesis of AF. Our group compared IAB in 144 patients with moderate-severe OSA (mean AHI = 56.2 ± 27.9) and 36 patients with mild or no OSA (mean AHI = 5.6 ± 3.6) and demonstrated that IAB was more prevalent in the moderate-severe OSA group with incidences of 34.7% versus 0%, respectively (*P* < 0.0001) [36]. The electrical remodeling leading to IAB may be mechanically mediated through intrathoracic pressure changes or a result of persistently elevated sympathetic tone [36-38]. This altered autonomic tone is also thought to have potential proarrhythmic effects on the PV ostia, as they are densely innervated by both vagal and adrenergic neurons [24].

A more recent study by Dimitri *et al* investigated 40 patients undergoing ablation of paroxysmal AF, 20 of which had OSA with an AHI ≥15 and 20 of which had an AHI < 15 [58]. They found that patients with OSA had prolonged conduction times along the coronary sinus and right atrium (p = 0.02), longer P-wave duration (p = 0.01), longer corrected sinus node recovery time (p = 0.02), lower atrial voltage (p < 0.001), and slower atrial conduction velocity (right atrium, p = 0.001, left atrium, p = 0.02). This suggests that OSA is associated with significant atrial remodeling characterized by atrial enlargement, reduction in voltage, as well as site-specific and widespread conduction abnormalities.

### Inflammation

C-reactive protein (CRP) has been demonstrated to be a sensitive marker for systemic inflammation and increased cardiovascular risk [59]. A study by Shamsuzzaman *et al* [60] reported an increase in CRP in patients with OSA that was proportional to the severity of OSA and independent of confounding disease states including obesity. Meanwhile, Chung *et al* [61] demonstrated an association between elevated CRP and AF by showing that CRP was more than 2-fold higher in AF patients when compared with controls, and that CRP was higher in the subgroup of patients with persistent versus paroxysmal AF. This suggests a possible association between OSA, inflammation, and AF. Though the mechanism underlying this observation has yet to be determined, there may be an association of elevated CRP with volume overloaded states, which in turn associated with both AF and OSA [62].

As can be seen, there are multiple plausible pathophysiologic mechanisms that may ultimately contribute to the association of OSA and AF, though further understanding of these mechanisms is necessary before one can conclusively point to a causal relationship. 

## EFFECTS OF OSA THERAPY ON AF

As has been elaborated upon thus far, there is growing evidence of plausible pathophysiologic explanations for the association of OSA and AF. Many recent studies have now turned to answer the ensuing question of whether treating OSA reduces the prevalence of AF. 

Early investigations of OSA treatment and the prevalence of cardiac arrhythmias utilized tracheostomy to alleviate upper airway obstruction. Tilkian *et al* [63] studied individuals with OSA for the presence of cardiac arrhythmias using several 24-hour Holter recordings pre- and post-tracheostomy and found a number of arrhythmias that were reversed post-tracheostomy, though AF was not mentioned among these arrhythmias. A larger study by Guillminault *et al* [21] assessed 50 subjects with OSA (AHI ≥ 25) peritracheostomy for the presence of cardiac arrhythmias. They noted frequent arrhythmias including episodes of sinus arrest, second degree AV block, PVCs, extreme sinus bradycardia as well as nocturnal paroxysmal AF in ten patients. With the exception of frequent PVCs, all the aforementioned arrhythmias had resolved when reassessed post-tracheostomy [21].

Continuous positive airway pressure (CPAP) therapy is now the mainstay of treatment of moderate-to-severe OSA, and may significantly decrease the risk of cardiovascular disease [64,65]. In fact, studies involving OSA patients with CHF have shown that chronic use of nocturnal CPAP therapy improves left ventricular systolic function [66-68], decreases sympathetic activity [66], reduces systolic blood pressure [67] and improves quality of life [66]. CPAP therapy studies involving OSA patients without CHF have also shown significant improvements in ventricular structural and functional changes including decreased ventricular wall thickness, decreased right ventricular dilation and improved contractility [69,70]. Even the acute administration of CPAP may have profound cardiovascular effects. Several studies have reported that acute CPAP administration decreases stroke volume and cardiac output in patients with CHF and OSA [71,72], and one recent study found similar decreases in stroke volume and cardiac output in a range of patients including OSA patients without CHF, post-operative cardiac surgery patients, as well as healthy volunteers [73]. However, these findings have not been consistently reported [74,75] and the acute cardiovascular effects of CPAP are not fully understood. 

It has been hypothesized that most of the benefit of CPAP therapy in OSA patients is due to the prevention of both the large negative swings in intrathoracic pressure (which increase left ventricular after load) and the associated arterial oxygen desaturation [76]. As many of these cardiac effects have been postulated to play a role in AF, it is not surprising that there is mounting evidence to suggest that CPAP can reduce the frequency of AF [77]. 

In fact, a recent study of a large Japanese population of 1,394 subjects identified that patients with OSA (AHI ≥ 5) had a higher prevalence of paroxysmal AF (1% for mild OSA, 3.3% for moderate OSA and 3.4% for severe OSA; p for trend = 0.051), and of 316 patients that underwent CPAP therapy, a significant reduction in the occurrence of paroxysmal AF was observed (n = 16 before CPAP versus n = 1 after CPAP; p < 0.001) [78]. Furthermore, a recent large observational intervention study by Kanagala *et al* assessed the effect of treatment of CPAP use in recurrence of AF after cardioversion in OSA patients. They found that untreated OSA (AHI ≥ 5) is associated with an 82% risk of recurrence of AF after successful cardioversion, while the control group had a 53% risk of recurrence (p = 0.009) [32]. Meanwhile, patients with OSA who were effectively treated with CPAP had a 42% risk of recurrence of AF after cardioversion. The reduced risk of recurrence in treated OSA patients compared to the control group may possibly be explained by undetected OSA in some of the control group patients. Multivariate analysis revealed that the risk of AF recurrence was related to the duration of nocturnal oxygen saturation <90% and the magnitude of desaturation. In a similar study of AF recurrence, Patel *et al* studied 3000 consecutive patients undergoing pulmonary vein antrum isolation as treatment for AF, 640 of which had OSA [79]. At the end of a follow-up period of 32 +/- 14 months, 78% of non-OSA patients were free of AF, versus only 73% of the OSA group (p = 0.024). OSA patients not treated with CPAP had higher early recurrence rates of AF than the CPAP group (55% versus 33%, p = 0.019) and, at the end of the follow-up period, 79% of the CPAP treated patients remained free of AF versus 68% of the non-CPAP patients [79].

The effects of CPAP in OSA patients may even extend as far as affecting the atrial electrical remodeling that is known to be associated with the disease [80]. It has been reported that there is an increase in maximum P-wave duration by up to 7.6 ms and an increase in P-wave dispersion (14.6 +/- 7.5 versus 8.9 +/- 3.1 ms, p < 0.001) in moderate-severe OSA patients compared to controls, highlighting the atrial electrical remodeling associated with the disease [55]. However, until now there has been very little as far as studies assessing the potential for CPAP to reverse these changes. One recent study investigated the effect of CPAP treatment on signal-averaged P-wave (SAPW) duration, which is an accepted marker for atrial electrical remodeling. It reports a shortening of SAPW duration (131.9 +/- 10.4 ms to 126.2 +/- 8.8 ms, p < 0.001) after four to six weeks of CPAP treatment in 19 severe OSA patients. This is thought to indicate improved atrial conduction and reverse electrical modeling after treatment with CPAP [81].

The reported changes in atrial electrical remodeling seen after treatment with CPAP may be partially attributable to the effects of CPAP on the left atrium. Oliveira *et al *found that OSA patients who received CPAP treatment for 24 weeks demonstrated a significantly increased left atrial passive emptying fraction (28.8 to 46.8%; p = 0.01) and a decreased left atrial active emptying fraction (42.7 to 25.7%; p <0.01) compared to those who received sham CPAP [82]. Another study demonstrated an increase in left atrial volume in CPAP-noncompliant patients (15.5 ± 22.3 mL; p <0.006) [83] and a potential protective effect of CPAP in the prevention of left atrial structural remodeling. 

The evidence thus far seems to suggest that relief of the airway obstruction that leads to nocturnal desaturations in OSA patients may result in improvement or reversal of cardiac arrhythmias, including AF [84]. CPAP, being the mainstay of OSA treatment has the largest body of evidence supporting this potential role, and it appears that it may reduce the frequency of AF by modifying some the physiologic factors thought to promote this arrhythmia including sympathetic activation, systemic inflammation, hypoxemia, cardiac dysfunction, and hypertension [38]. Even so, there remains insufficient evidence to conclusively delineate the exact role of CPAP in the treatment of AF in OSA patients and further investigations with randomized trials are required. 

## CONCLUSION

As can be seen, there is mounting evidence of a strong association between OSA and AF. Whether SBD, in particular OSA, leads to AF is an appealing but currently unproven hypothesis. Controversy remains as to whether OSA is a primary etiologic factor for AF because of the high incidence of cardiovascular comorbidities in persons diagnosed with OSA. While there has been significant progress in separating individual risk factors for AF and OSA, they are complex disease processes with numerous dynamic interactions. Meanwhile, the potential pathophysiologic associations continue to be investigated and the treatment effects of OSA on development of AF continue to be explored.
